# Timely Sharing of Data on Infection and Death of Medical Workers

**DOI:** 10.3389/fpubh.2020.552409

**Published:** 2020-10-21

**Authors:** Weipu Mao, Ming Chen

**Affiliations:** Department of Urology, Affiliated Zhongda Hospital of Southeast University, Nanjing, China

**Keywords:** COVID-19, medical workers, infection, death, overwork

## Introduction

Since December 2019, an outbreak of a new type of coronavirus pneumonia (COVID-19) has attracted worldwide attention. COVID-19 has rapidly evolved into a global pandemic that has required urgent action from the international community (1). The epidemic has now spread all over the world, with the number of confirmed cases and deaths still rising. As of 2:00 a.m. CEST, August 1, 2020, there have been 17,406,428 confirmed cases worldwide, of which 669,740 have died (2).

## Baseline Characteristics of Death Medical Workers in China, The United States and Italy

According to incomplete statistics from official and public reports (3, 4), as of August 1, 2020, at least 64 medical workers in China have given their lives whilst working to prevent and control COVID-19, compared to at least 851 in the United States (of which 156 have complete clinical data), and these numbers continue to increase (Supplementary Table 1). The virus has resulted in the death of a higher proportion of male medical workers in both China (79.7%) and the United States (55.8%). In China, the most deceased medical workers were between 50 and 59 years old (Supplementary Figures 1A,B), accounting for ~54%, with a median age of 50 years (48.4 ± 12.8 years). Among both male and female workers in the United States, the age group with the highest number of deaths among medical workers was 60–69 years old (Supplementary Figures 1A,B), about 35.3%, with a median age of 57 years (55.2 ± 12.8 years). In addition, nearly half of Chinese medical workers died of other diseases due to overwork, 24 (38.7%) died from COVID-19 infection, and eight died from accidents (Supplementary Table 1).

According to statistics, the peak months for medical workers' deaths were February and April in China and the United States, respectively (Supplementary Figures 1C,D). In China, most of the medical worker deaths were doctors (37, 57.8%), while in the United States, nurses accounted for the majority of deaths among medical workers (75, 48.1%). In both China and the United States, medical worker deaths were more concentrated in the hardest-hit areas (New York and New Jersey in the United States and Hubei Province in China).

In China, the peak of medical worker deaths occurred ~1 week after the highest number of daily new cases, whereas in the United States and other countries such as Italy, the number of medical worker deaths has increased since the COVID-19 pandemic (Supplementary Figures 1C,D). In the United States, it appears that the number of medical worker deaths ceased to increase in June, but the above data do not include the deaths of medical workers due to incomplete clinical data, overwork, and other accidental factors.

## Discussion

Compared with the current basic stability of the epidemic in China, the situation abroad is still relatively serious. In the United States, Brazil and India, more than 1.5 million cases have been confirmed, especially in the United States, where the number of confirmed cases has exceeded 4 million. Moreover, the number of new confirmed cases in India is increasing exponentially. In some states of the United States, medical personnel account for as many as 20% of known coronavirus cases (4). With the increasing severity of the epidemic, the number of confirmed cases has skyrocketed, and the workload, working hours, and burden of medical workers have also increased (5, 6). In addition, infections among medical workers are also a worrying problem (7). With the increasing number of COVID-19 patients, there is a severe shortage of personal protective equipment for medical workers, including masks, goggles, protective clothing, and so on. Due to the severe shortage of protective equipment, medical workers are forced to reuse disposable masks and homemade protective clothing, which is still facing a huge risk of infection.

There are also serious issues concerning the infection of medical workers in Spain. According to the Spanish Ministry of Health, as of April 6, 2020, ~19,400 medical workers were confirmed to be infected with COVID-19, accounting for about 14% of the confirmed cases. Of these, 13 medical workers have died, including 11 doctors, 1 nurse and 1 health worker.

In the United States, as of August 1, 2020, there were 11 emergency medical technicians among deceased medical workers. These first-responders work in ambulance and emergency medical services and were often the first medical professionals to have contact with patients with COVID-19. Infections of these personnel may therefore be due to a failure to take adequate preventive measures and insufficient protection in the epidemic (8). First-line emergency personnel need clearer measures and to be given adequate protective equipment.

With the intensification of the pandemic, medical health care systems across the world continue to overloaded. The sacrifice of medical workers reminds governments that they need to invest in more medical resources, strengthen the protection of medical workers, give priority to ensuring the supply of resources to front-line medical workers, and take good care of the physical and mental health of these workers by reducing stress. This may involve more rational arrangements for the rotation of shifts and transfer of staff to allow for rest and to enhance medical confidence and resistance to the epidemic (9). The timely sharing of data on the infection and related deaths of medical workers by governments could enable us to better understand and assess the risks faced by medical workers, enabling us to provide real-time guidance, early intervention and better responses to the COVID-19 pandemic.

## Author Contributions

WM and MC drafted the manuscript, studied the concept, and design. WM collected and analyzed the data. All authors contributed to the article and approved the submitted version.

## Conflict of Interest

The authors declare that the research was conducted in the absence of any commercial or financial relationships that could be construed as a potential conflict of interest.

## Supplementary Material

Supplementary Figure 1 Distribution of deceased medical workers in China, the United States and Italy. **(A)** pie chart of age distribution; **(B)** histogram of age distribution of different genders; **(C)** Daily new cases; **(D)** Daily deaths of medical workers.

Supplementary Table 1 Baseline characteristics of death medical workers in China, the United States and Italy^a^.
