# Supplementary figures and images for: Timely Sharing of Data on Infection and Death of Medical Workers
Source: Front Public Health. 2020 Oct 21;8:552409. doi: 10.3389/fpubh.2020.552409 (PMC7609716; doi:10.3389/fpubh.2020.552409)

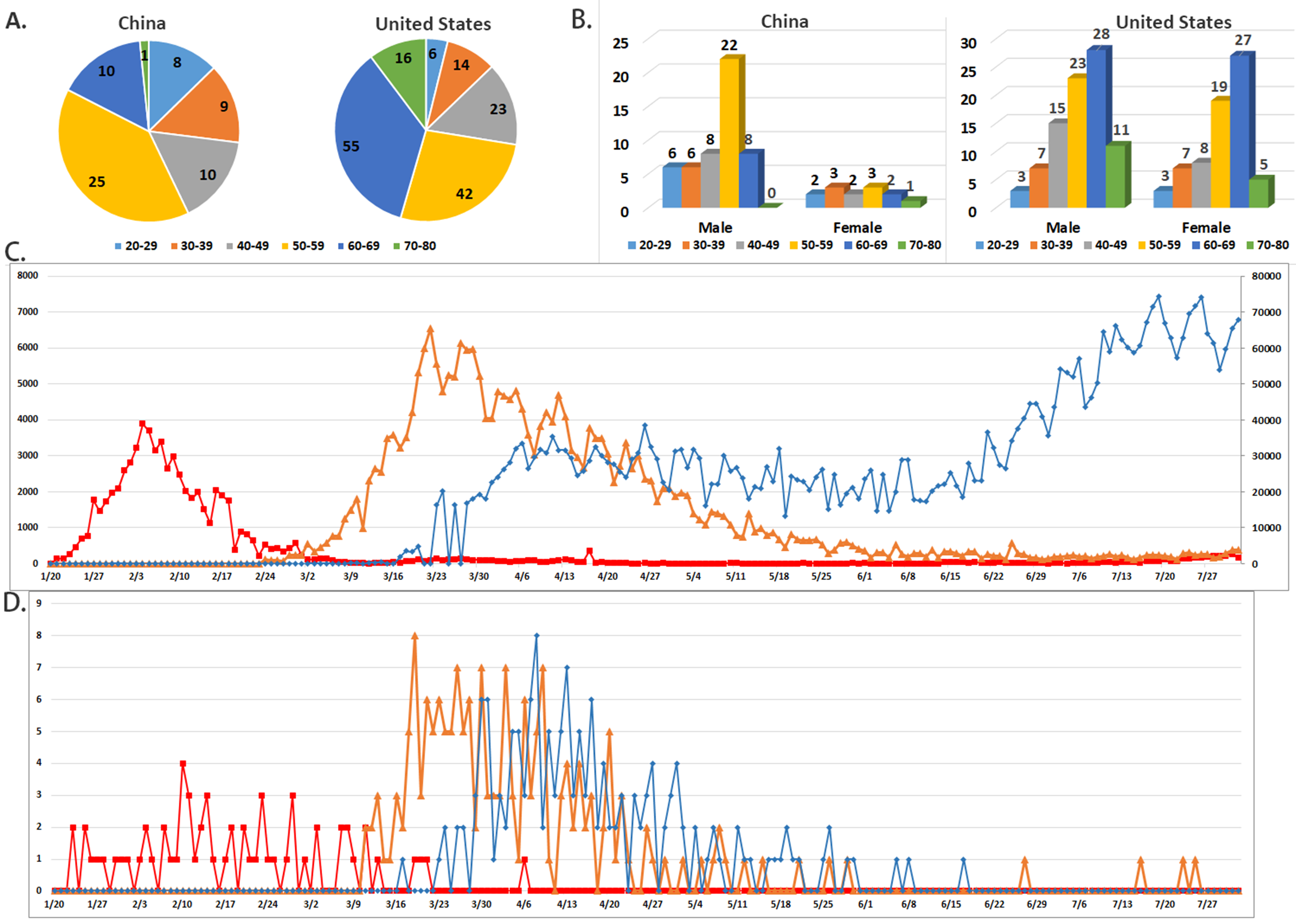

Supplement: Supplementary Figure 1 — Distribution of deceased medical workers in China, the United States and Italy. (A) pie chart of age distribution; (B) histogram of age distribution of different genders; (C) Daily new cases; (D) Daily deaths of medical workers. [file Figure_1.TIF]
