# Supplementary material for: Timely Sharing of Data on Infection and Death of Medical Workers
Source: Front Public Health. 2020 Oct 21;8:552409. doi: 10.3389/fpubh.2020.552409 (PMC7609716; doi:10.3389/fpubh.2020.552409)
Supplement: Supplementary Table 1 — Baseline characteristics of death medical workers in China, the United States and Italya. [file Table_1.DOCX]

**Table 1. Baseline characteristics of death medical workers in China, the United States and Italy ^a^.**

| Characteristics | China, N (%) | United States, N (%) | Italy, N (%) |
| --- | --- | --- | --- |
| All patients | 64 | 156 | 176 |
| Age [years] ^a^ |  |  |  |
| Median (IQR) | 50 (37-57) | 57 (48-64) | - |
| Mean ± standard | 48.4±12.8 | 55.2±12.8 | - |
| 20-29 | 8 (12.5) | 6 (3.8) | - |
| 30-39 | 9 (14.1) | 14 (9.0) | - |
| 40-49 | 10 (15.6) | 23 (14.7) | - |
| 50-59 | 25 (39.1) | 42 (26.9) | - |
| 60-69 | 10 (15.6) | 55 (35.3) | - |
| 70-80 | 1 (1.6) | 16 (10.3) | - |
| Sex |  |  |  |
| Male | 51 (79.7) | 87 (55.8) | - |
| Female | 13 (20.3) | 69 (44.2) | - |
| Causes |  |  |  |
| COVID-19 | 24 (38.7) | 156 (100.0) | 176 (100.0) |
| Overwork | 30 (48.4) | - | - |
| Accident | 8 (12.9) | - | - |
| Month |  |  |  |
| January | 9 (14.3) | - | - |
| February | 39 (61.9) | - | - |
| March | 14 (22.2) | 23 (14.7) | 88 (50.0) |
| April | 1 (1.6) | 102 (65.4) | 73 (41.5) |
| May | - | 28 (17.9) | 11 (6.3) |
| June | - | 3 (1.9) | 1 (0.6) |
| July |  |  | 3 (1.7) |
| Occupation |  |  |  |
| Doctor | 37 (57.8) | 24 (15.4) | - |
| Nurse | 2 (3.1) | 75 (48.1) | - |
| Others ^b^ | 25 (39.1) | 57 (36.5) | - |
| State |  |  |  |
| New Jersey | - | 28 (17.9) | - |
| New York | - | 37 (23.7) | - |
| Others ^c^ | - | 91 (58.3) | - |
| Origin |  |  |  |
| Hubei Province | 34 (53.1) | - | - |
| Non-Hubei Province | 30 (46.9) | - | - |

**Abbreviations:**

IQR, Interquartile range.

**Note:** 2 Chinese medical workers death cause and 1 Chinese medical worker death time were missing. ^a^ Data as of August 1, 2020. ^b^ Including paramedics and crucial health care support staff (including 1 Chinese emergency medical technician, 10 American emergency medical technicians).
